# Supplementary material for: Stereomeric Lipopeptides from a Single Non-Ribosomal Peptide Synthetase as an Additional Source of Structural and Functional Diversification in Pseudomonas Lipopeptide Biosynthesis
Source: Int J Mol Sci. 2023 Sep 19;24(18):14302. doi: 10.3390/ijms241814302 (PMC10531924; doi:10.3390/ijms241814302)
Supplement: Supplementary file 1 [file ijms-24-14302-s001.zip › ijms-2576713-supplementary.pdf]

Supplementary Materials for

Stereomeric Lipopeptides from a Single Non-Ribosomal Peptide Synthetase as an Additional Source of Structural and Functional Diversification in *Pseudomonas* Lipopeptide Biosynthesis

Penthip Muangkaew [1], Vic De Roo [1,2], Lu Zhou [3], Léa Girard [4], Catherine Cesa-Luna [4], Monica Höfte [3], René De Mot [4], Annemieke Madder [1], Niels Geudens [2,*] and José C. Martins [2,*]

[1] Organic and Biomimetic Chemistry Research Group, Department of Organic and Macromolecular Chemistry, Faculty of Science, Ghent University, B-9000 Ghent, Belgium
[2] NMR and Structure Analysis Unit, Department of Organic and Macromolecular Chemistry, Faculty of Science, Ghent University, B-9000 Ghent, Belgium
[3] Laboratory of Phytopathology, Department of Plants and Crops, Faculty of Bioscience Engineering, Ghent University, B-9000 Ghent, Belgium
[4] Centre of Microbial and Plant Genetics, Faculty of Bioscience Engineering, Katholieke Universiteit Leuven, Heverlee, Belgium
* Correspondence: niels.geudens@ugent.be (N.G.); jose.martins@ugent.be (J.C.M.)

This file includes:

- Figures S1 to S22
- Tables S1 to S4
- Scheme S1

Table of Contents

Supplementary data..4

Biosynthesis and characterization of natural entolysins 4

Pseudomonas entomophila COR5...4

Pseudomonas entomophila L48..10

Pseudomonas sp. COR16 11

Pseudomonas sp. COR17 12

Pseudomonas sp. COW47..12

Pseudomonas sp. COR6...13

Chemical synthesis of the entolysin analogues 15

Supplementary references...18

Figures

Figure S1: Semi-preparative reversed-phase HPLC chromatogram of the crude extract from P. entomophila COR5. A and B indicate the presence of major (Entolysin A) and minor products (Entolysin B) respectively..4

Figure S2: ^{1}H-^{13}C HSQC fingerprint region of entolysin A. (500 MHz, DMF-d7, 298K)...........................4

Figure S3: ^{1}H-^{13}C HSQC fingerprint region of entolysin B. (500 MHz, DMF-d7, 298K)...........................4

Figure S4: Combined chemical-shift perturbations for entolysin A vs entolysin B..............................7

Figure S5: Prediction of epi-inactive E/C-domain in second module. 8

Figure S6: Expanded view of the main C2* cluster (red) including EtlA C2 (green). 9

Figure S7: Semi-preparative reversed-phase HPLC chromatogram of the crude extract from P. entomophila L48. A and B indicate the presence of major (Entolysin A) and minor products (Entolysin B) respectively..10

Figure S8: NMR fingerprint matching of entolysin A from P. entomophila COR5 (black) vs P. entomophila L48 (blue). (500 MHz, DMF-d7, 298K)...10

Figure S9: NMR fingerprint matching of entolysin B from P. entomophila COR5 (black) vs P. entomophila L48 (blue). (500 MHz, DMF-d7, 298K) 10

Figure S10: Semi-preparative reversed-phase HPLC chromatogram of the crude extract from Pseudomonas sp. COR16. A and B indicate the presence of major (Entolysin A) and minor products (Entolysin B) respectively...11

Figure S11: NMR fingerprint matching of entolysin A from P. entomophila COR5 (black) vs Pseudomonas sp. COR16 (blue). (500 MHz, DMF-d7, 298K) 11

Figure S12: NMR fingerprint matching of entolysin B from P. entomophila COR5 (black) vs Pseudomonas sp. COR16 (blue). (500 MHz, DMF-d7, 298K) 11

Figure S13: Semi-preparative reversed-phase HPLC chromatogram of the crude extract from Pseudomonas sp. COR17. A and B indicate the presence of major (Entolysin A) and minor products (Entolysin B) respectively...12

Figure S14: Semi-preparative reversed-phase HPLC chromatogram of the crude extract from Pseudomonas sp. COW47. A and B indicate the presence of major (Entolysin A) and minor products (Entolysin B) respectively...12

Figure S15: NMR fingerprint matching of entolysin A from P. entomophila COR5 (black) vs Pseudomonas sp. COW47 (blue). (500 MHz, DMF-d7, 298K)...13

Figure S16: NMR fingerprint matching of entolysin B from P. entomophila COR5 (black) vs Pseudomonas sp. COW47 (blue). (500 MHz, DMF-d7, 298K) ... 13
Figure S17: HPLC chromatogram of the extract from Pseudomonas sp. COR6. ... 13
Figure S18: NMR fingerprint matching of entolysin A from P. entomophila COR5 (black) vs Pseudomonas sp. COR6 (blue). (500 MHz, DMF-d7, 298K) ... 14
Figure S19: NMR fingerprint matching of entolysin B from P. entomophila COR5 (black) vs Pseudomonas sp. COR6 (blue). (500 MHz, DMF-d7, 298K) ... 14
Figure S20 LCMS chromatogram of the pure l-Ser13 (1). Kinetex C18 column with linear gradient over 6 min of 5 mM NH_4OAc in H_2O and CH_3CN from 50% to 100% CH_3CN. ... 17
Figure S21 LCMS chromatogram of the pure l-Ser10 (2). Kinetex C18 column with linear gradient over 6 min of 5 mM NH_4OAc in H_2O and CH_3CN from 50% to 100% CH_3CN. ... 18
Figure S22. LCMS chromatogram of the pure d-Ser10-d-Ser13 (3). Kinetex C18 column with linear gradient over 6 min of 5 mM NH_4OAc in H_2O and CH_3CN from 50% to 100% CH_3CN. ... 18

Tables

Table S1: NMR assignment (^1H and ^{13}C) of entolysin A, extracted as a major compound produced by P. entomophila COR5. (500MHz, DMF-d7, 298K) ... 5
Table S2: NMR assignment (^1H and ^{13}C) of entolysin B, extracted as a minor compound produced by P. entomophila COR5. (500MHz, DMF-d7, 298K) ... 6
Table S3: Analytical HPLC data of entolysin A after performing Marfey's analysis ... 7
Table S4: Analytical HPLC data of entolysin B after performing Marfey's analysis ... 7

Scheme

Scheme S1 Synthetic route towards synthetic Entolysin B analogues (1-3). Reagents and conditions: a) (i) 20% piperidine, DMF; (ii) 5eq of 0.5M Fmoc-AA-OH, 0.5M Oxyma pure in DMF, 0.5M DIC in NMP; b) Alloc-L-Leu-OH, DIC, DMAP, DMF, 37 °C, 24h; c) $[Pd(PPh_3)_4]$, $PhSiH_3$ ... 15

Supplementary data

Biosynthesis and characterization of natural entolysins

Pseudomonas entomophila COR5

Purification

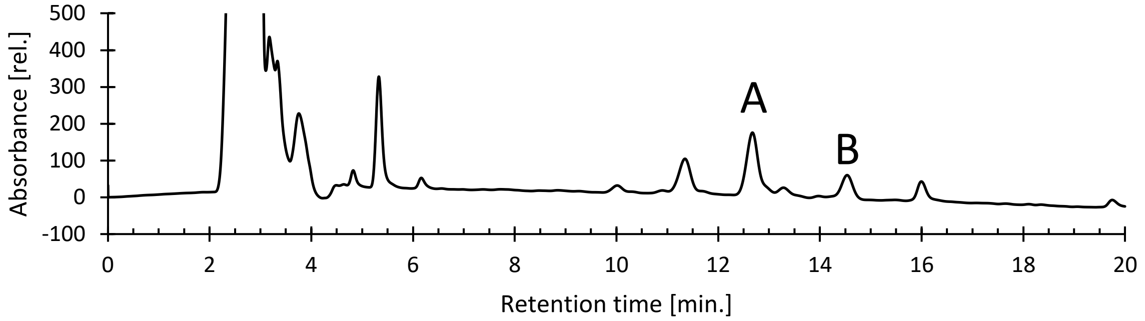

Figure S1: Semi-preparative reversed-phase HPLC chromatogram of the crude extract from P. entomophila COR5. A and B indicate the presence of major (Entolysin A) and minor (Entolysin B) products, respectively.

NMR analyses

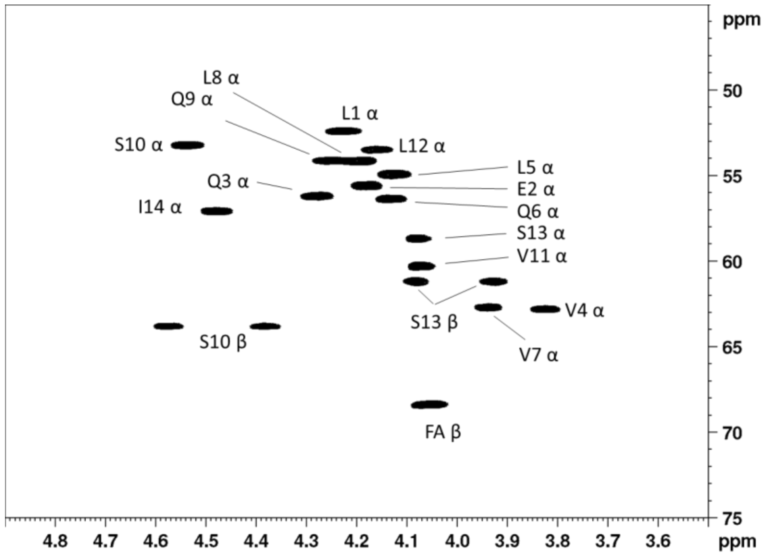

Figure S2: ^{1}H-^{13}C HSQC fingerprint region of entolysin A (500 MHz, DMF-d7, 298K).

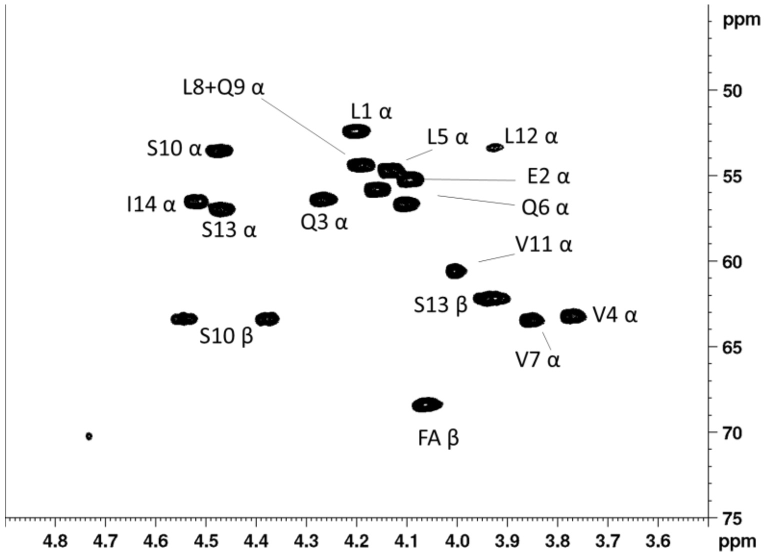

Figure S3: ^{1}H-^{13}C HSQC fingerprint region of entolysin B (500 MHz, DMF-d7, 298K).

Table S1. NMR assignment (^{1}H and ^{13}C) of entolysin A, extracted as a major compound produced by P. entomophila COR5 (500MHz, DMF-d7, 298K).

			^{1}H δ	^{13}C δ
**HDA**				
		CO	-	173.07
		CH$_2$α	2.45	43.68
		CHβ	4.05	68.38
		CH$_2$γ	1.48	37.59
		CH$_2$δ1	1.47	25.56
		CH$_2$δ2	1.35	25.56
		CH$_2$ε	1.28	29.30
		CH$_2$ζ	1.29	29.65
		CH$_2$η	1.27	31.83
		CH$_2$θ	1.29	22.53
		CH$_2$ι	0.88	13.72
		OH	n.d.	-
**Leu1**				
$^3J_{HNH\alpha}$	6.1 Hz	NH	8.63	-
		CHα	4.21	52.37
		CO	-	175.03
		CH$_2$β	1.72	39.46
		CHγ	1.75	24.57
		CH$_3$δ1	0.95	22.81
		CH$_3$δ2	0.91	21.36
**Glu2**				
$^3J_{HNH\alpha}$	5.15 Hz	NH	8.87	-
		CHα	4.17	55.65
		CO	-	174.71
		CH$_2$β1	2.11	26.25
		CH$_2$β2	2.06	26.23
		CH$_2$γ	2.51	30.27
		COδ	-	n.d.
		OH	n.d.	-
**Gln3**				
$^3J_{HNH\alpha}$	5.6 Hz	NH	8.45	-
		CHα	4.27	56.24
		CO	-	n.d.
		CH$_2$β	2.16	26.50
		CH$_2$γ1	2.45	32.15
		CH$_2$γ2	2.39	32.08
		COδ	-	n.d.
		NH2	7.54/6.92	-
**Val4**				
$^3J_{HNH\alpha}$	5.9 Hz	NH	7.81	-
		CHα	3.81	62.89
		CO	-	n.d.
		CHβ	2.30	29.19
		CH$_1$γ1	1.07	20.00
		CH$_2$γ2	0.98	18.75
**Leu5**				
$^3J_{HNH\alpha}$	5.15 Hz	NH	7.86	-
		CHα	4.12	54.97
		CO	-	175.47
		CH$_2$β1	1.86	39.11
		CH$_2$β2	1.64	39.11
		CHγ	1.82	26.61
		CH$_3$δ1	0.94	22.51
		CH$_3$δ2	0.89	20.98
**Gln6**				
$^3J_{HNH\alpha}$	4.7 Hz	NH	8.10	-
		CHα	4.12	56.41
		CO	-	n.d.
		CH$_2$β1	2.31	26.73
		CH$_2$β2	2.12	26.73
		CH$_2$γ1	2.52	32.10
		CH$_2$γ2	2.31	32.10
		COδ	-	n.d.
		NH2	7.35/6.83	-
**Val7**				
$^3J_{HNH\alpha}$	n.d.	NH	7.75	-
		CHα	3.92	62.74
		CO	-	n.d.
		CHβ	2.33	29.30
		CH$_1$γ1	1.11	19.72
		CH$_2$γ2	0.99	19.10
**Leu8**				
$^3J_{HNH\alpha}$	n.d.	NH	7.99	-
		CHα	4.18	54.18
		CO	-	n.d.
		CH$_2$β1	1.94	39.61
		CH$_2$β2	1.57	39.61
		CHγ	1.95	24.36
		CH$_3$δ1	0.92	22.93
		CH$_3$δ2	0.88	20.51
**Gln9**				
$^3J_{HNH\alpha}$	n.d.	NH	7.75	-
		CHα	4.25	54.12
		CO	-	n.d.
		CH$_2$β1	2.23	26.84
		CH$_2$β2	2.16	26.84
		CH$_2$γ1	2.45	31.91
		CH$_2$γ2	2.33	31.91
		COδ	-	n.d.
		NH2	7.30/6.79	-
**Ser10**				
$^3J_{HNH\alpha}$	6.5 Hz	NH	7.96	-
		CHα	4.53	53.25
		CO	-	n.d.
		CH$_2$β1	4.58	63.81
		CH$_2$β2	4.38	63.81
		OH	n.d.	-
**Val11**				
$^3J_{HNH\alpha}$	n.d.	NH	7.68	-
		CHα	4.07	60.29
		CO	-	n.d.
		CHβ	2.23	29.86
		CH$_1$γ1	1.05	18.21
		CH$_2$γ2	1.03	18.99
**Leu12**				
$^3J_{HNH\alpha}$	n.d.	NH	7.99	-
		CHα	4.16	53.46
		CO	-	n.d.
		CH$_2$β1	1.86	39.40
		CH$_2$β2	1.76	39.40
		CHγ	1.67	24.94
		CH$_3$δ1	0.92	23.20
		CH$_3$δ2	0.91	21.00
**Ser13**				
$^3J_{HNH\alpha}$	6.7 Hz	NH	7.94	-
		CHα	4.08	58.66
		CO	-	n.d.
		CH$_2$β1	4.08	61.18
		CH$_2$β2	3.93	61.18
		OH	n.d.	-
**Ile14**				
$^3J_{HNH\alpha}$	n.d.	NH	7.67	-
		CHα	4.47	57.06
		CO	-	n.d.
		CHβ	2.02	36.92
		CH$_2$γ1	1.56	24.99
		CH$_2$γ2	1.23	24.99
		CH$_3$γ	0.91	15.49
		CH$_3$δ2	0.87	11.08

Table S2. NMR assignment (^1H and ^{13}C) of entolysin B, extracted as a minor compound produced by P. entomophila COR5 (500MHz, DMF-d7, 298K).

				^1H δ	^{13}C δ
**HDA**					
			CO	-	n.d.
			CH_2α	2.46	43.65
			CHβ	4.07	68.35
			CH_2γ	1.48	37.56
			CH_2δ1	1.46	25.54
			CH_2δ2	1.34	25.54
			CH_2ε	1.28	29.28
			CH_2ζ	1.29	29.66
			CH_2η	1.27	31.85
			CH_2θ	1.28	22.51
			CH_2ι	0.88	13.72
			OH	n.d.	-
**Leu1**					
	$^3J_{HNH\alpha}$	6.4 Hz	NH	8.65	
			CHα	4.20	52.34
			CO	-	n.d.
			CH_2β	1.73	39.50
			CHγ	1.74	24.54
			CH_3δ1	0.95	22.76
			CH_3δ2	0.91	21.37
**Glu2**					
	$^3J_{HNH\alpha}$	4.9 Hz	NH	8.88	-
			CHα	4.16	55.77
			CO	-	n.d.
			CH_2β1	2.11	26.14
			CH_2β2	2.06	26.14
			CH_2γ	2.51	30.20
			COδ	-	n.d.
			OH	n.d.	-
**Gln3**					
	$^3J_{HNH\alpha}$	5.6 Hz	NH	8.46	-
			CHα	4.27	56.35
			CO	-	n.d.
			CH_2β	2.17	26.41
			CH_2γ1	2.45	32.17
			CH_2γ2	2.40	32.13
			COδ	-	n.d.
			NH2	7.54/6.92	-
**Val4**					
	$^3J_{HNH\alpha}$	5.8 Hz	NH	7.82	-
			CHα	3.77	63.19
			CO	-	n.d.
			CHβ	2.31	29.09
			CH_1γ1	1.08	20.16
			CH_2γ2	0.98	18.67
**Leu5**					
	$^3J_{HNH\alpha}$	5.1 Hz	NH	7.86	-
			CHα	4.10	55.20
			CO	-	n.d.
			CH_2β1	1.87	38.98
			CH_2β2	1.64	38.98
			CHγ	1.82	24.55
			CH_3δ1	0.94	22.49
			CH_3δ2	0.89	21.05
**Gln6**					
	$^3J_{HNH\alpha}$	4.7 Hz	NH	8.13	-
			CHα	4.10	56.65
			CO	-	n.d.
			CH_2β1	2.32	26.59
			CH_2β2	2.13	26.59
			CH_2γ1	2.51	32.08
			CH_2γ2	2.31	32.08
			COδ	-	n.d.
			NH2	7.35/6.83	-

				^1H δ	^{13}C δ
**Val7**					
	$^3J_{HNH\alpha}$	5.7 Hz	NH	7.80	-
			CHα	3.85	63.43
			CO	-	n.d.
			CHβ	2.34	29.32
			CH_1γ1	1.13	20.11
			CH_2γ2	0.99	19.05
**Leu8**					
	$^3J_{HNH\alpha}$	n.d.	NH	8.02	-
			CHα	4.13	54.69
			CO	-	n.d.
			CH_2β1	1.96	39.61
			CH_2β2	1.54	39.61
			CHγ	1.97	24.33
			CH_3δ1	0.92	23.30
			CH_3δ2	0.88	20.52
**Gln9**					
	$^3J_{HNH\alpha}$	6.9 Hz	NH	7.69	-
			CHα	4.19	54.35
			CO	-	n.d.
			CH_2β1	2.14	26.98
			CH_2β2	2.14	26.98
			CH_2γ1	2.47	31.92
			CH_2γ2	2.33	31.92
			COδ	-	n.d.
			NH2	7.30/6.79	-
**Ser10**					
	$^3J_{HNH\alpha}$	n.d.	NH	7.77	-
			CHα	4.48	53.48
			CO	-	n.d.
			CH_2β1	4.55	63.35
			CH_2β2	4.38	63.35
			OH	n.d.	-
**Val11**					
	$^3J_{HNH\alpha}$	4.1 Hz	NH	7.88	-
			CHα	4.00	60.54
			CO	-	n.d.
			CHβ	2.23	29.66
			CH_1γ1	1.10	18.33
			CH_2γ2	1.08	18.82
**Leu12**					
	$^3J_{HNH\alpha}$	n.d.	NH	8.02	-
			CHα	3.93	53.31
			CO	-	n.d.
			CH_2β1	2.03	38.19
			CH_2β2	1.87	38.19
			CHγ	1.69	24.96
			CH_3δ1	0.92	23.23
			CH_3δ2	0.92	20.47
**Ser13**					
	$^3J_{HNH\alpha}$	n.d.	NH	7.77	-
			CHα	4.47	56.98
			CO	-	n.d.
			CH_2β1	3.93	62.09
			CH_2β2	3.93	62.09
			OH		
**Ile14**					
	$^3J_{HNH\alpha}$	9.6 Hz	NH	7.52	-
			CHα	4.53	56.48
			CO	-	n.d.
			CHβ	1.99	36.73
			CH_2γ1	1.54	24.90
			CH_2γ2	1.20	24.90
			CH_3γ	0.90	15.75
			CH_3δ2	0.85	11.21

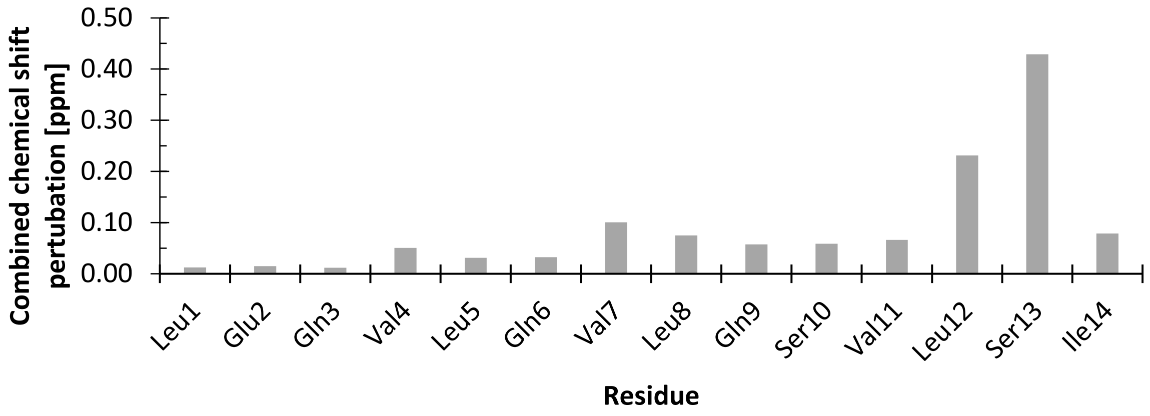

Figure S4. Combined chemical-shift perturbations for entolysin A vs entolysin B.

Marfey's analysis on entolysin A

Table S3. Analytical HPLC data of entolysin A after performing Marfey's analysis showing the occurrence of individual D- and L-amino acids.

		RT (min)	Area	Area%	Height	Height%	Ratio
Leu	D	42,4	1207,8	2,8	106,0	3,8	2D-2L
	L	37,6	957,7	2,2	86,7	3,1	
Ser	D	19,3	456,3	1,0	52,5	1,9	1D-1L
	L	17,9	373,3	0,9	40,9	1,5	
Val	D	39,6	2010,2	4,6	137,4	4,9	2D-1L
	L	32,3	687,1	1,6	30,8	1,1	
Glx	D	25,1	318,0	0,7	8,3	0,3	All D
	L	Peak absent					
Ile	D	Peak absent					1L
	L	35,7	946,9	2,2	92,5	3,3	

Marfey's analysis on entolysin B

Table S4. Analytical HPLC data of entolysin B after performing Marfey's analysis showing the occurrence of individual D- and L-amino acids.

		RT (min)	Area	Area%	Height	Height%	Ratio
Leu	D	42,4	1483,0	1,3	125,4	2,5	2D-2L
	L	37,3	2041,0	1,8	281,5	3,4	
Ser	D	20,0	1312,3	1,1	117,8	2,3	2D
	L	Peak absent					
Val	D	37,6	3268,5	2,8	154,8	2,1	2D-1L
	L	32,3	445,4	0,5	27,3	0,7	
Glx	D	25,1	7370,1	6,3	435,2	8,5	All D
	L	Peak absent					
Ile	D	Peak absent					1L
	L	36,71	968,74	1,15	70,84	1,85	

Phylogenetic analysis

Prediction of epi-inactive E/C-domain at module 2

Phylogenetic analysis of currently available E/C-classified domains, experimentally classified as either epi-active or epi-inactive, was performed as described in [1]. Other C-domains, previously classified as C_{start} (C1 or Ci) and $^{L}C_{L}$, are not included in this analysis. Epi-inactive E/C-type domains are labeled with an asterisk (Cn*, with n referring to the consecutive module number). The epi-inactive C2*-domains of different lipopeptide NRPS systems are found in a few (sub)clusters and highlighted in red. Other epi-inactive domains are shown in blue. The entolysin-associated domains are shown in green. The C2-domain of entolysin NRPS EtlA (strains L48 and COR5) is located in the main red cluster comprising C2* for biosynthesis of xantholysin (most related one), but also for C2* of the Viscosin family members (WLIP, viscosin, viscosinamide, massetolide, pseudodesmin, pseudophomin), poaeamide and orfamide. This strongly suggests that the entolysin C2-domain would also lack epimerizing activity.

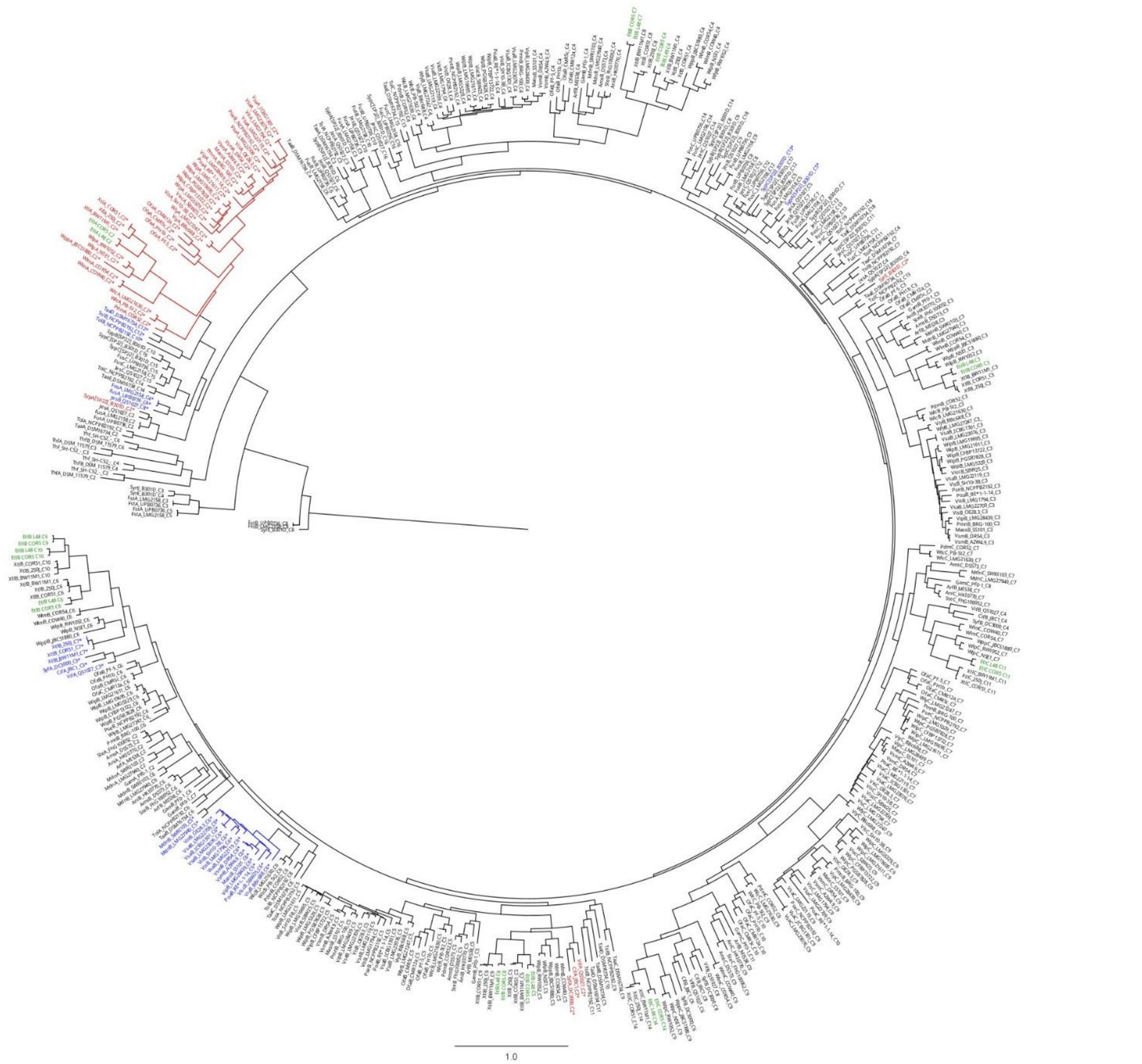

Figure S5. Prediction of epi-inactive E/C-domain in the second module of the NRPS systems of Pseudomonas entomophila COR5.

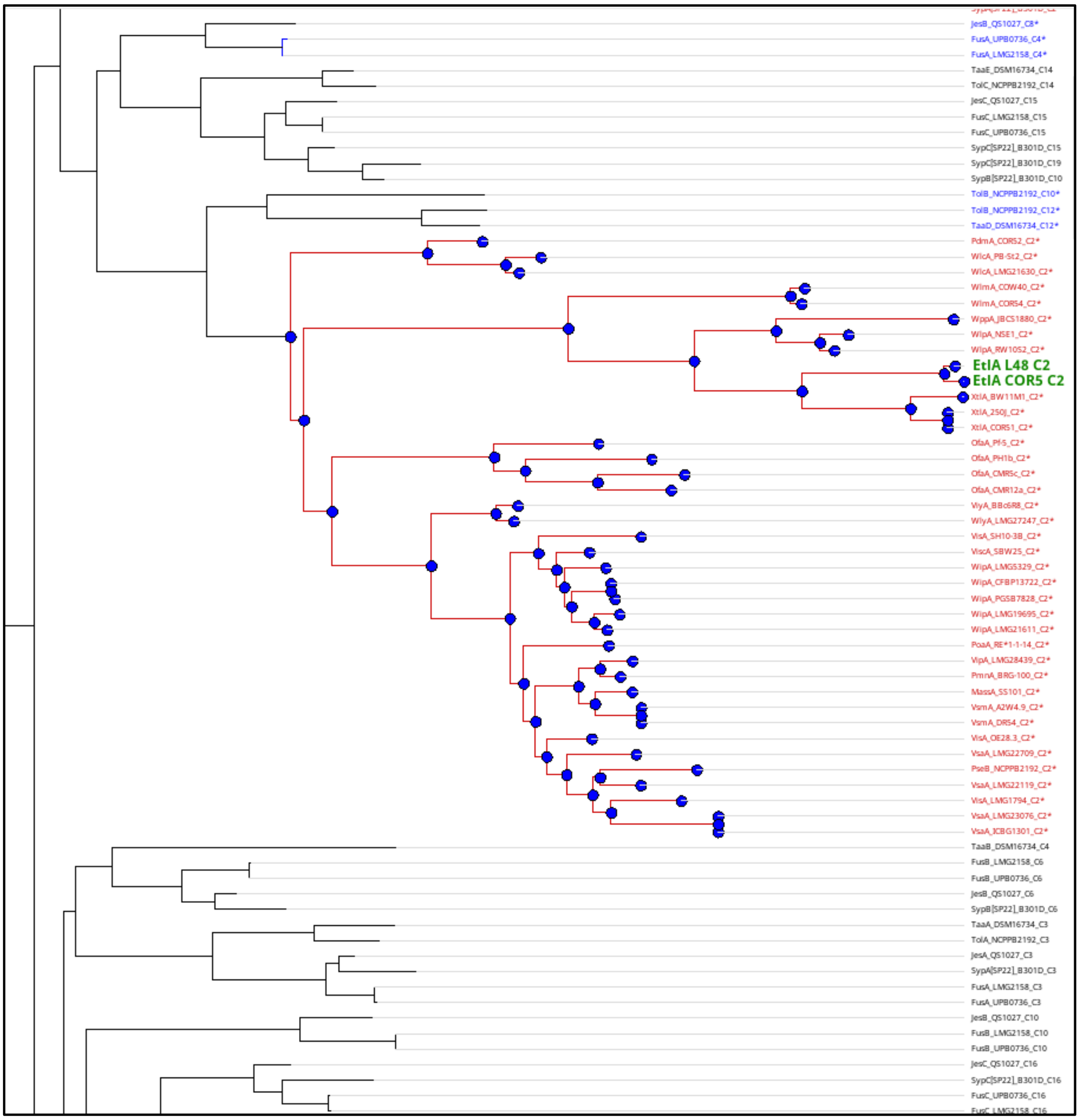

Figure S6. Expanded view of the main C2 cluster (red) including EtlA C2 (green).*

Pseudomonas entomophila L48

Chromatogram

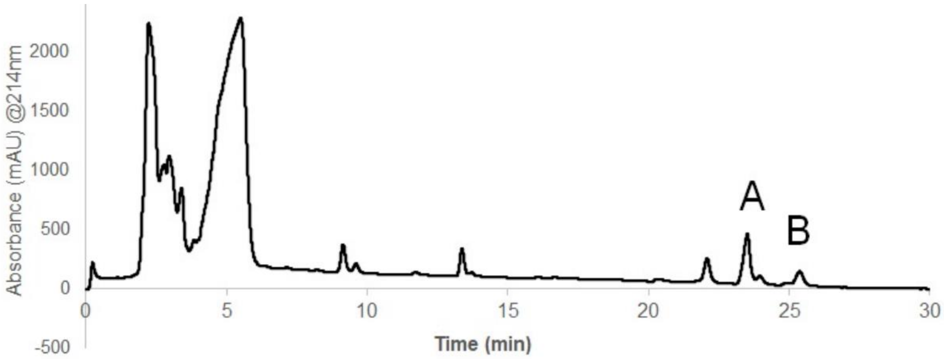

Figure S7. Semi-preparative reversed-phase HPLC chromatogram of the crude extract from P. entomophila L48. A and B indicate the presence of major (Entolysin A) and minor (Entolysin B) products, respectively.

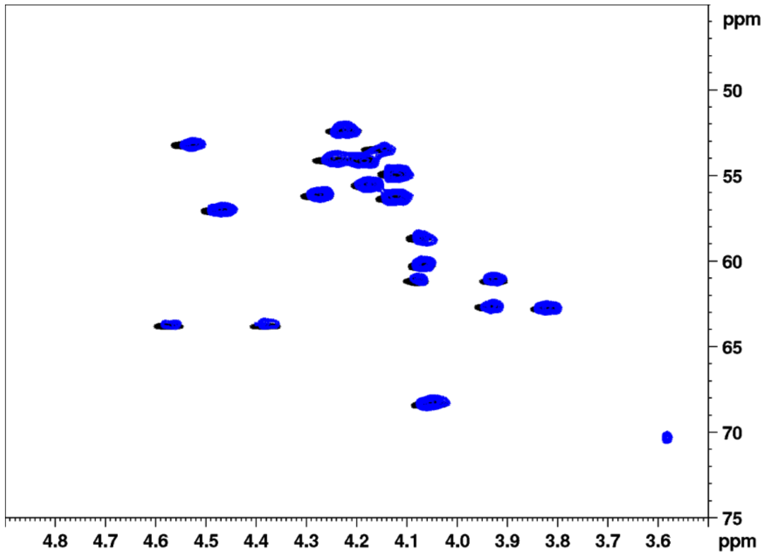

Figure S8. NMR fingerprint matching of entolysin A from P. entomophila COR5 (black) vs P. entomophila L48 (blue) (500 MHz, DMF-d7, 298K).

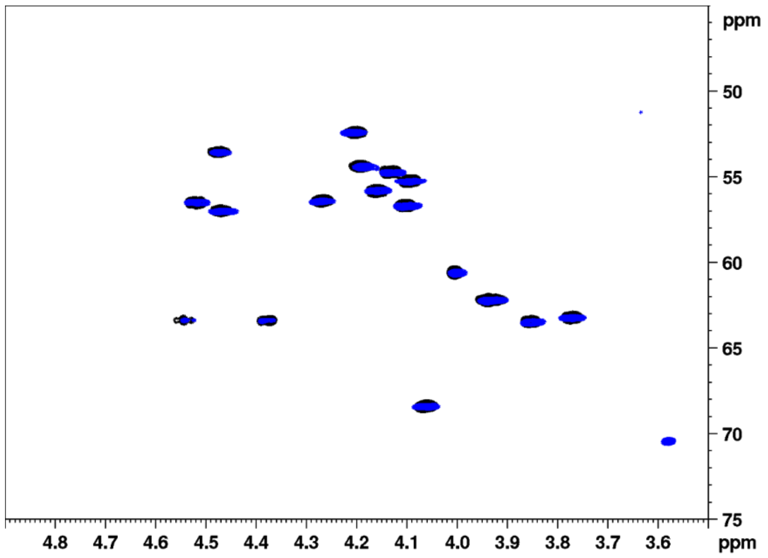

Figure S9. NMR fingerprint matching of entolysin B from P. entomophila COR5 (black) vs P. entomophila L48 (blue) (500 MHz, DMF-d7, 298K).

Pseudomonas sp. COR16

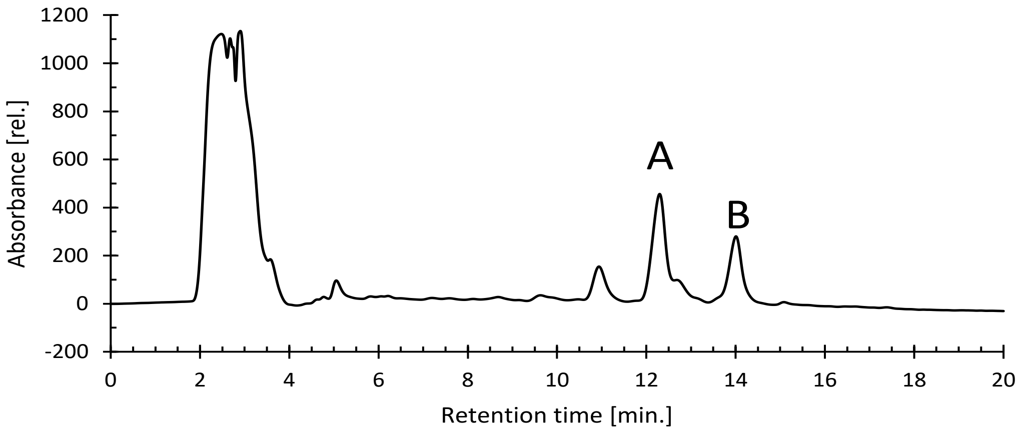

Figure S10. Semi-preparative reversed-phase HPLC chromatogram of the crude extract from Pseudomonas sp. COR16. A and B indicate the presence of major (Entolysin A) and minor (Entolysin B) products, respectively.

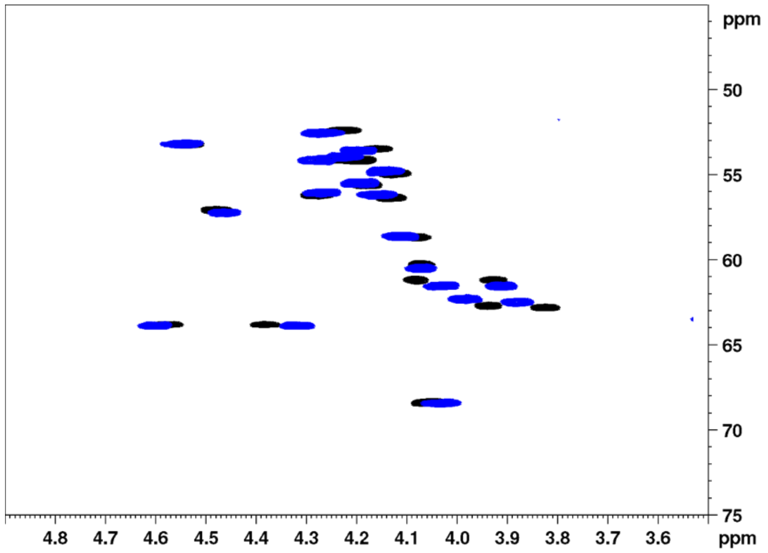

Figure S11. NMR fingerprint matching of entolysin A from P. entomophila COR5 (black) vs Pseudomonas sp. COR16 (blue) (500 MHz, DMF-d7, 298K).

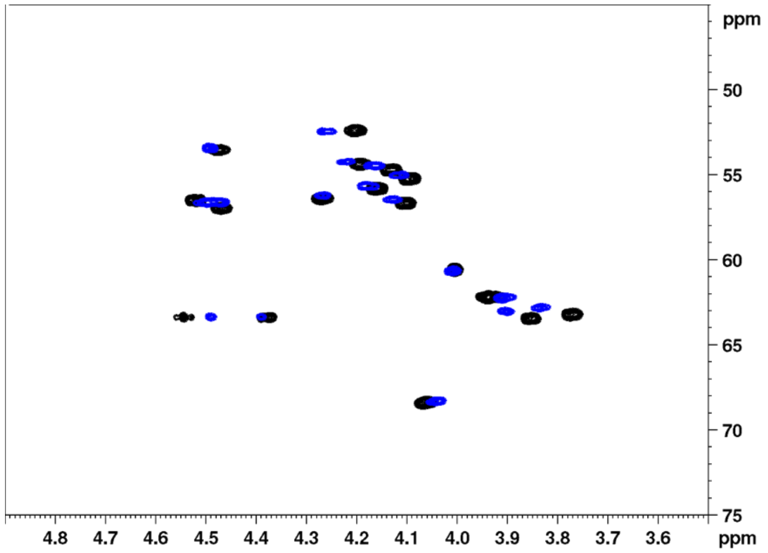

Figure S12. NMR fingerprint matching of entolysin B from P. entomophila COR5 (black) vs Pseudomonas sp. COR16 (blue) (500 MHz, DMF-d7, 298K).

Pseudomonas sp. COR17

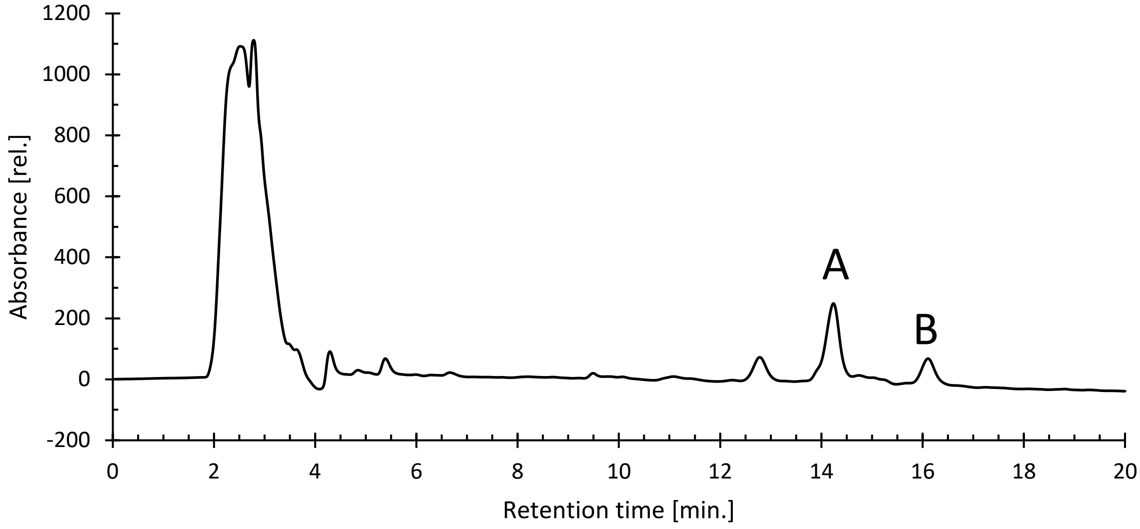

Figure S13. Semi-preparative reversed-phase HPLC chromatogram of the crude extract from Pseudomonas sp. COR17. A and B indicate the presence of major (Entolysin A) and minor (Entolysin B) products, respectively.

Pseudomonas sp. COW47

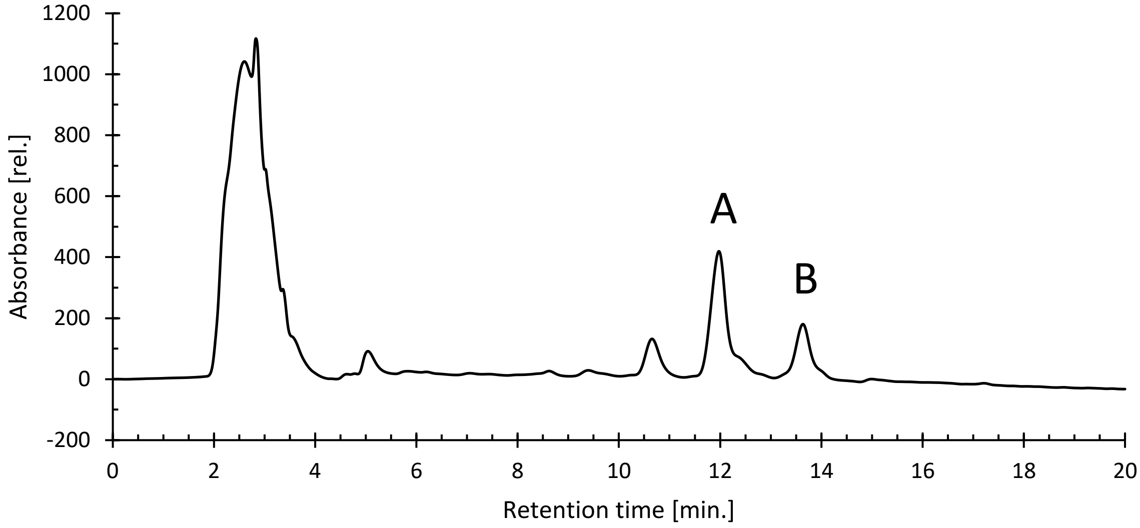

Figure S14. Semi-preparative reversed-phase HPLC chromatogram of the crude extract from Pseudomonas sp. COW47. A and B indicate the presence of major (Entolysin A) and minor (Entolysin B) products, respectively.

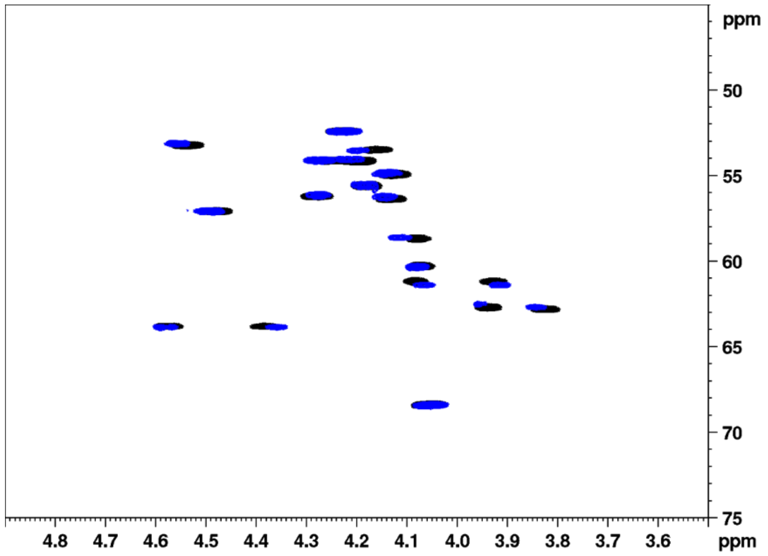

Figure S15. NMR fingerprint matching of entolysin A from P. entomophila COR5 (black) vs Pseudomonas sp. COW47 (blue) (500 MHz, DMF-d7, 298K).

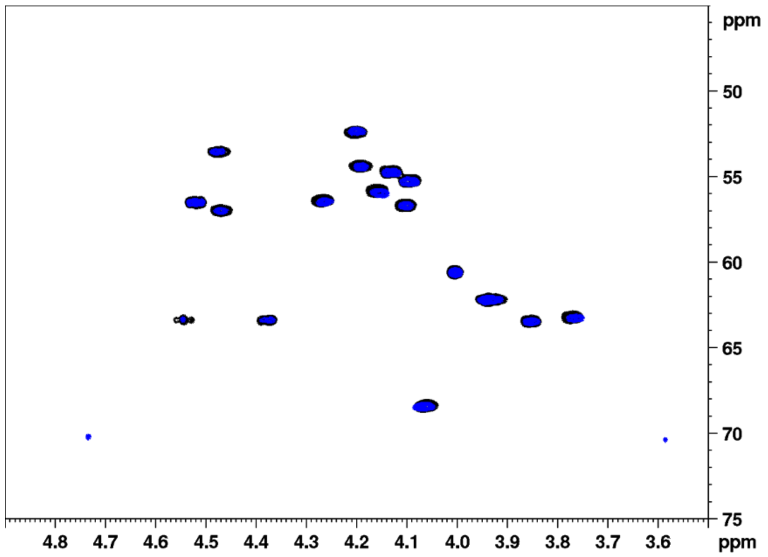

Figure S16. NMR fingerprint matching of entolysin B from P. entomophila COR5 (black) vs Pseudomonas sp. COW47 (blue) (500 MHz, DMF-d7, 298K).

Pseudomonas sp. COR6

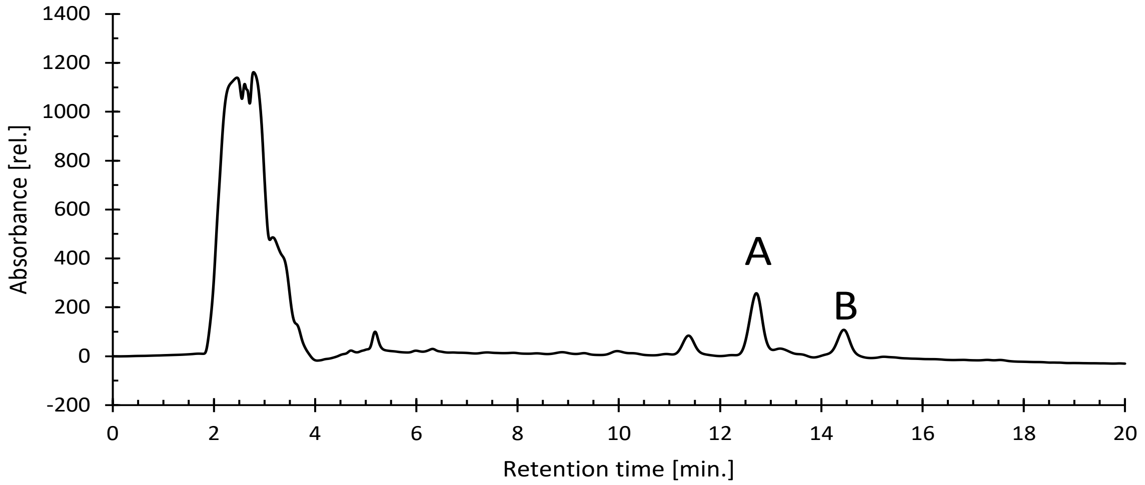

Figure S17. HPLC chromatogram of the extract from Pseudomonas sp. COR6.

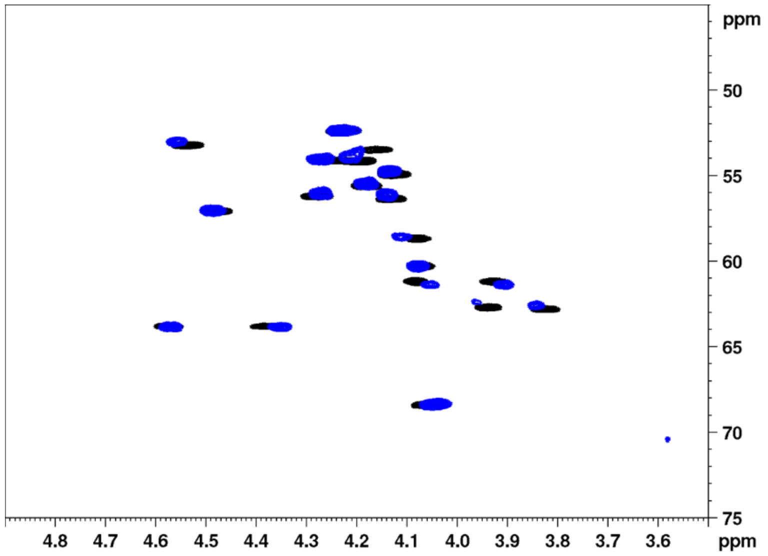

Figure S18. NMR fingerprint matching of entolysin A from P. entomophila COR5 (black) vs Pseudomonas sp. COR6 (blue) (500 MHz, DMF-d7, 298K).

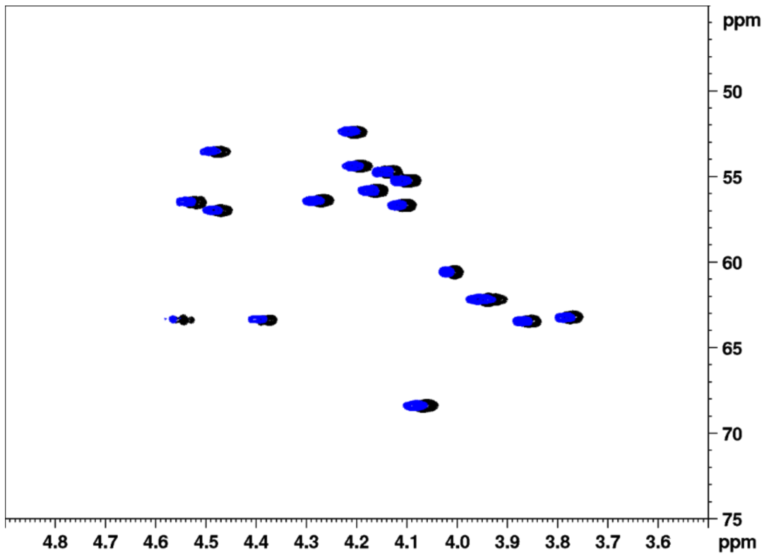

Figure S19. NMR fingerprint matching of entolysin B from P. entomophila COR5 (black) vs Pseudomonas sp. COR6 (blue) (500 MHz, DMF-d7, 298K).

Chemical synthesis of the entolysin analogues

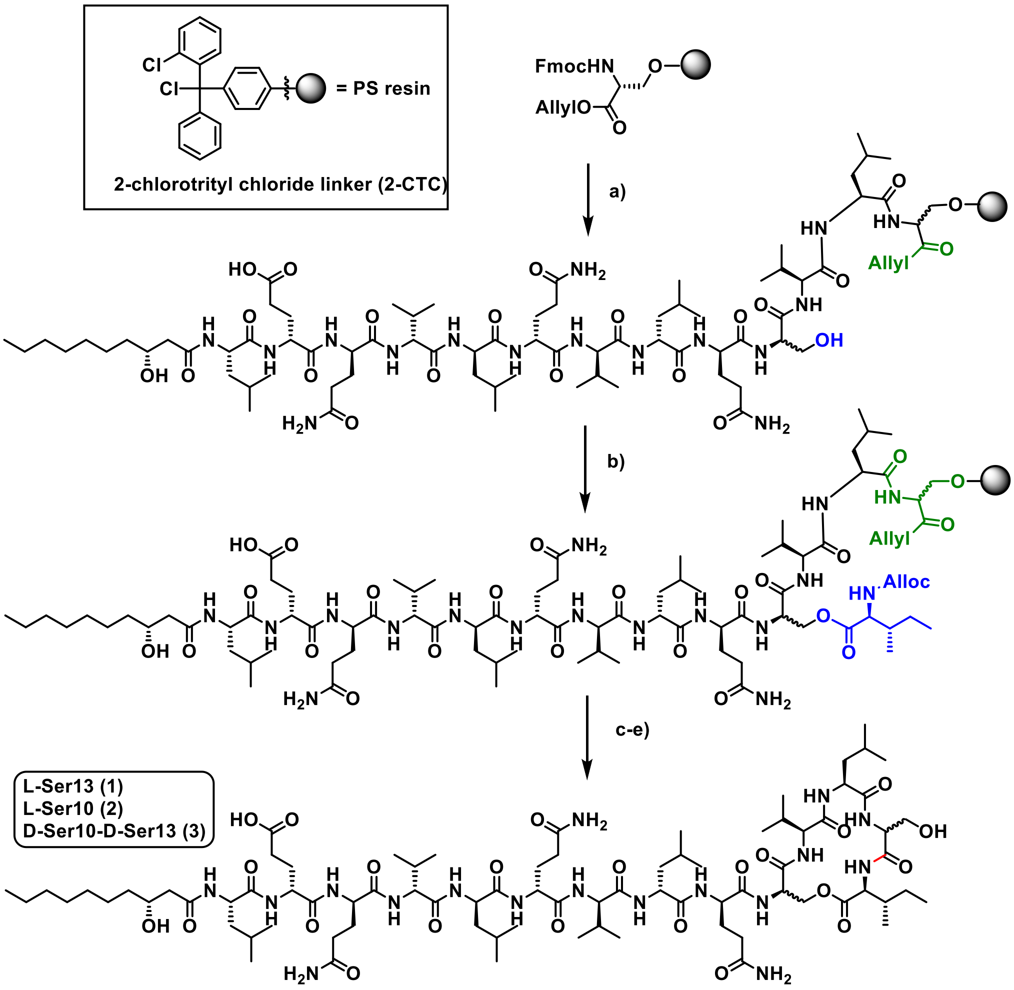

Scheme S1. Synthetic route towards synthetic Entolysin B analogues (1-3). Reagents and conditions: a) (i) 20% piperidine, DMF; (ii) 5eq of 0.5M Fmoc-AA-OH, 0.5M Oxyma pure in DMF, 0.5M DIC in NMP; b) Alloc-L-Leu-OH, DIC, DMAP, DMF, 37 °C, 24h; c) [Pd(PPh_3)$_4$], $PhSiH_3$.

Loading of the 2-CTC resin via side-chain anchoring of Fmoc-D-Ser(OH)-OAll

Fmoc-D-Ser(OH)-OAll (1.75 g, 4.76 mmol, 1.5 eq.) was dissolved in dry DMF in a dry pear-shaped flask under an inert (Ar) atmosphere. Subsequently, dry pyridine (769 µL, 9.51 mmol, 3eq.) was added dropwise. This reaction mixture was brought into a dry reaction vessel (100 ml.), where the 2-CTC resin (1.60 mmol/g, 1.98 g, 3.17 mmol, 1eq.) was located. The reactor was placed in a Selecta Vibromatic shaker and connected to a thermostat with a temperature set at 60°C, and the reactor was shaken overnight (24 h). Next, the excess reagents were filtered off, and the resin was washed with dry DCM. Unreacted functionalities were capped by adding a DCM:MeOH:DIPEA (15 ml, 17:2:1, 2×10 min.) solution. After washing with DCM (3x), DMF (3x) and DCM (3x), the beads were dried on the oil pump overnight before loading determination. The loading was determined by UV monitoring the dibenzofulvene-piperidine adduct formed after Fmoc-deprotection and loading of 0.514 mmol/g was obtained.

Loading of the 2-CTC resin via side-chain anchoring of Fmoc-L-Ser(OH)-OAll

Fmoc-L-Ser(OH)-OAll (1.75 g, 4.76 mmol, 1.5 eq.) was dissolved in dry DMF in a dry pear-shaped flask under an inert (Ar) atmosphere. Subsequently, dry pyridine (769 µL, 9.51 mmol, 3eq.) was added dropwise. This reaction mixture was brought into a dry reaction vessel (100 ml.), where the 2-CTC resin (1.60 mmol/g, 1.98 g, 3.17 mmol, 1eq.) was located. The reactor was placed in a Selecta

Vibromatic shaker and connected to a thermostat with a temperature set at 60°C, and the reactor was shaken overnight (24h). Next, the excess of reagents was filtered off, the resin was washed with dry DCM, and unreacted functionalities were capped by adding a DCM:MeOH:DIPEA (15 ml, 17:2:1, 2×10 min.) solution. After washing with DCM (3x), DMF (3x) and DCM (3x), the beads were dried on the oil pump overnight before loading determination. The loading was determined by UV monitoring the dibenzofulvene-piperidine adduct formed after Fmoc-deprotection and loading of 0.732 mmol/g was obtained.

Solid-phase peptide synthesis towards L-Ser13 (1)

Automated solid-phase peptide synthesis was performed on a INTAVIS apparatus. In a C→N fashion, Fmoc-L-Leu-OH, Fmoc-L-Val-OH, Fmoc-D-Ser(OH)-OH, Fmoc-D-Gln(Trt)-OH, Fmoc-D-Leu-OH, Fmoc-D-Val-OH, Fmoc-D-Gln(Trt), Fmoc-D-Leu-OH, Fmoc-D-Val-OH, Fmoc-D-Gln(Trt)-OH, Fmoc-D-Glu(OtBu)-OH and Fmoc-L-Leu-OH were coupled onto the modified Fmoc-L-Ser-OAll-2-CTC resin (68 mg, 50 µmol). The automated synthesis did not include the lipid tail ((*R*)-3-(tert-butyldimethylsilyloxy)decanoic acid). Then, the lipid tail was inserted manually after Fmoc deprotection of the last amino acid (L-Leu) on the solid support. The resin was washed thoroughly with the use of DMF (6x), DCM (3x) and MeOH (3x). The esterification was performed as follows: Alloc-L-Ile was dissolved with dry THF (1 mL) in a dry pear-shaped flask under an Ar atmosphere and was placed in the ice bath, DIC was added to the solution and stirred for 30 min, DMAP was dissolved in 500 µL of dry THF and transferred the solution to the reaction mixture (Ile and DIC), the preactivated solution was added to the peptidyl resin in the 1.5 mL Eppendorf, and the reaction was placed in the thermoshakers at 37 °C for 20h. The reaction needed to be repeated twice. The resin was washed thoroughly with the use of THF (6x), DMF (3x), DCM (3x). The cyclization was performed in the condition of 0.5M Oxymapure in DMF, 0.5M DIC in the presence of 0.05eq DIPEA in NMP at room temperature for 4h. The resin was washed thoroughly in the same manner as described previously. Afterwards, the completion of the reaction was checked by a small cleavage test, and the obtained peptide was subjected to LC-MS analysis.

Solid-phase peptide synthesis towards L-Ser10 (2)

Automated solid-phase peptide synthesis was performed using an INTAVIS apparatus. In a C→N fashion, Fmoc-L-Leu-OH, Fmoc-L-Val-OH, Fmoc-L-Ser(OH)-OH, Fmoc-D-Gln(Trt)-OH, Fmoc-D-Leu-OH, Fmoc-D-Val-OH, Fmoc-D-Gln(Trt), Fmoc-D-Leu-OH, Fmoc-D-Val-OH, Fmoc-D-Gln(Trt)-OH, Fmoc-D-Glu(OtBu)-OH and Fmoc-L-Leu-OH were coupled onto the modified Fmoc-D-Ser-OAll-2-CTC resin (97 mg, 50 µmol). The automated synthesis did not include the lipid tail ((R)-3-(tert-butyldimethylsilyloxy)decanoic acid). After Fmoc deprotection of the last amino acid (L-Leu) on the solid support, the lipid tail was manually inserted. The resin was thoroughly washed using DMF (6x), DCM (3x) and MeOH (3x). For esterification, Alloc-L-Ile was dissolved in dry THF (1 mL) in a dry pear-shaped flask under an Ar atmosphere and placed in an ice bath. DIC was added to the solution and stirred for 30 minutes. DMAP was dissolved in 500 µL of dry THF and then transferred to the reaction mixture (Ile and DIC). The preactivated solution was added to the peptidyl resin in a 1.5 mL Eppendorf tube, and the reaction was placed in a thermoshaker at 37°C for 20 hours. The resin was washed thoroughly using THF (6x), DMF (3x) and DCM (3x). Cyclization was performed under the following conditions: 0.5M Oxymapure in DMF, 0.5M DIC in the presence of 0.05 eq DIPEA in NMP at room temperature for 4 hours. The resin was again washed thoroughly as described previously. Afterwards, the completion of the reaction was checked by a small cleavage test, and the obtained peptide was subjected to LC-MS analysis."

Solid-phase peptide synthesis towards D-Ser10-D-Ser13 (3)

Automated solid-phase peptide synthesis was performed using an INTAVIS apparatus. In a C→N fashion, Fmoc-L-Leu-OH, Fmoc-L-Val-OH, Fmoc-D-Ser(OH)-OH, Fmoc-D-Gln(Trt)-OH, Fmoc-D-Leu-OH, Fmoc-D-Val-OH, Fmoc-D-Gln(Trt), Fmoc-D-Leu-OH, Fmoc-D-Val-OH, Fmoc-D-Gln(Trt)-OH, Fmoc-D-Glu(OtBu)-OH and Fmoc-L-Leu-OH were coupled onto the modified Fmoc-D-Ser-OAll-2-CTC resin (97 mg, 50 µmol). The automated synthesis did not include the lipid tail ((R)-3-(tert-butyldimethylsilyloxy)decanoic acid). Then, the lipid tail was inserted manually after Fmoc deprotection of the last amino acid (L-Leu) on the solid support. The resin was thoroughly washed with DMF (6x), DCM (3x) and MeOH (3x). For esterification, Alloc-L-Ile was dissolved in dry THF (1 mL) in a dry pear-shaped flask under an Ar atmosphere and placed in an ice bath. DIC was added to the solution and stirred for 30 minutes. DMAP was dissolved in 500 µL of dry THF and transferred to the reaction mixture (Ile and DIC). The preactivated solution was added to the peptidyl resin in a 1.5 mL Eppendorf tube, and the reaction was placed in the thermoshaker at 37°C for 20 hours. The resin was thoroughly washed with THF (6x), DMF (3x) and DCM (3x). Cyclization was performed under the following conditions: 0.5M Oxymapure in DMF, 0.5M DIC in the presence of 0.05 eq DIPEA in NMP at room temperature for 4 hours. The resin was washed thoroughly in the same manner as described previously. Afterwards, the completion of the reaction was checked by a small cleavage test, and the obtained peptide was subjected to LC-MS analysis.

LCMS of Synthetic Entolysin B analogues

L-Ser13 (1)

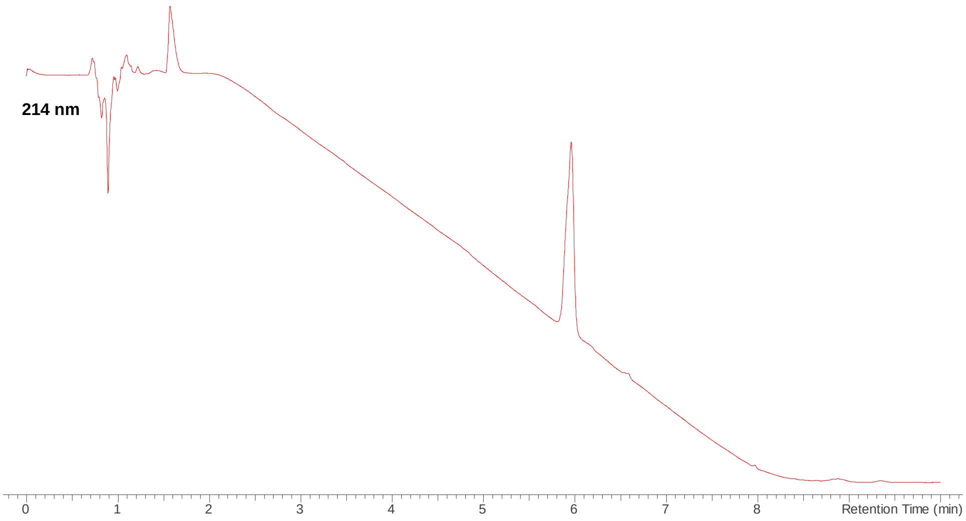

Figure S20. LCMS chromatogram of the pure l-Ser13 (1). Kinetex C18 column with linear gradient over 6 min of 5 mM NH_4OAc in H_2O and CH_3CN from 50% to 100% CH_3CN.

L-Ser10 (2)

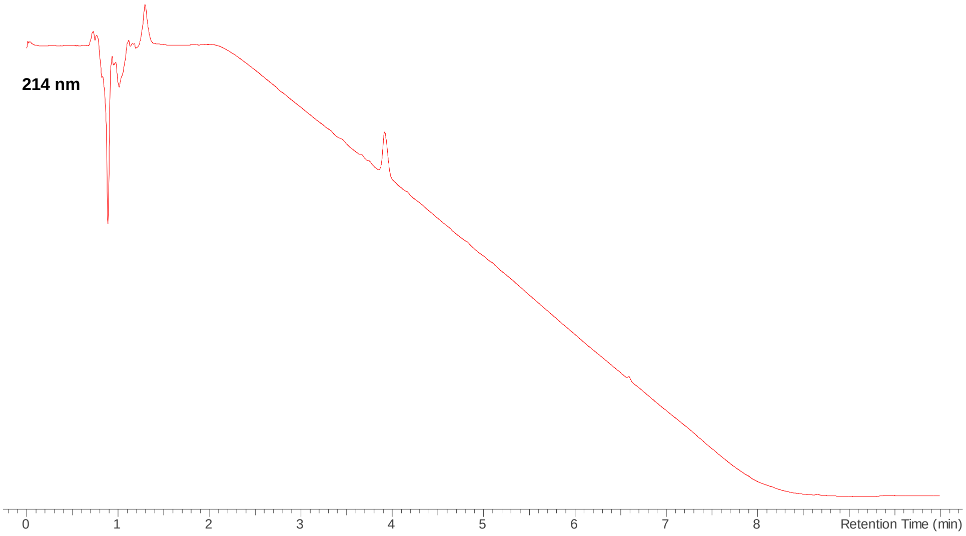

Figure S21. LCMS chromatogram of the pure l-Ser10 (2). Kinetex C18 column with linear gradient over 6 min of 5 mM NH_4OAc in H_2O and CH_3CN from 50% to 100% CH_3CN.

D-Ser10-D-Ser13 (3)

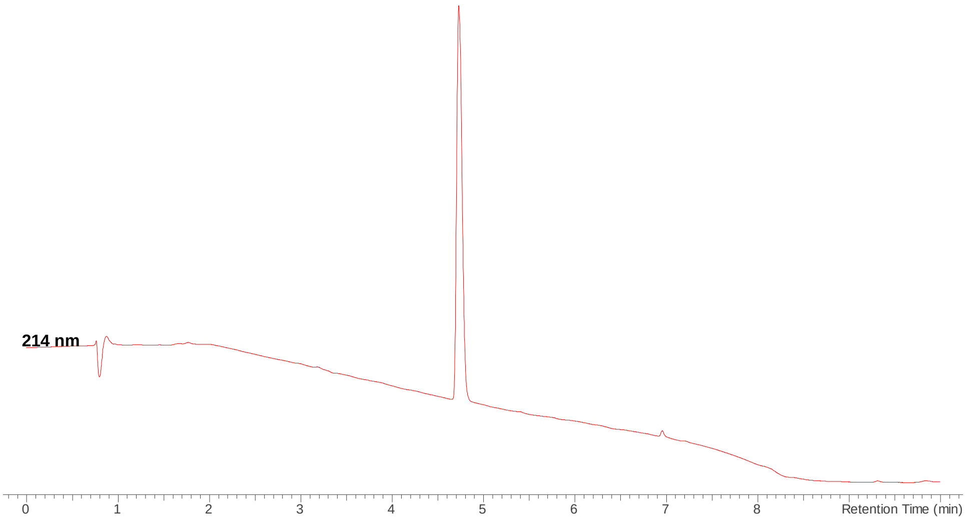

Figure S22. LCMS chromatogram of the pure d-Ser10-d-Ser13 (3). Kinetex C18 column with linear gradient over 6 min of 5 mM NH_4OAc in H_2O and CH_3CN from 50% to 100% CH_3CN.

Supplementary references

1. Cesa-Luna, C., et al., *Charting the lipopeptidome of nonpathogenic Pseudomonas.* mSystems, 2023: p. e00988-22.
